# Preliminary In-Situ Evaluation of an Innovative, Semi-Flexible Pavement Wearing Course Mixture Using Fast Falling Weight Deflectometer

**DOI:** 10.3390/ma11040611

**Published:** 2018-04-16

**Authors:** Chiara Pratelli, Giacomo Betti, Tullio Giuffrè, Alessandro Marradi

**Affiliations:** 1 Civil and Industrial Engineering Department, University of Pisa, 56122 Pisa, Italy; g.betti@dic.unipi.it (G.B.); a.marradi@ing.unipi.it (A.M.); 2 Faculty of Engineering and Architecture, University of Enna Kore, 94100 Enna, Italy; tullio.giuffre@unikore.it

**Keywords:** Accelerated Pavement Testing (APT), Fast Falling Weight Deflectometer (FFWD), semi-flexible pavement, Grouted Macadam, field-testing

## Abstract

In the last forty, years semi-flexible pavements have been successfully employed, especially in those areas subjected to heavy and slow-moving loads. They usually comprise a wearing course of Grouted Macadam, a composite pavement material that provides significant advantages in comparison to both concrete and asphalt pavements. On the other hand, the laying process of this material is a two-stage operation, and the realization complexity leads to long realization times and high initial costs. Therefore, the use of semi-flexible pavements has been limited to some fields of application and areas. Recently, an innovative material has been developed to be used as an alternative to Grouted Macadam for semi-flexible pavement wearing course realization. This material should provide similar or even superior characteristics compared to traditional Grouted Macadam. This will reduce semi-flexible pavement construction time and avoid the need for dividing the laying process. This paper presents an experimental program involving the use of FastFWD, as an APT device, to evaluate in-situ properties and performance of this material. The achieved results regarding the validation of this new material by means of FastFWD appear promising both in terms of the material’s properties and resistance to dynamic load repetitions.

## 1. Introduction

Road pavements are built up in several layers constructed from a wide variety of materials and mixtures. They are traditionally classified into three main categories, according to the building materials used as top layers and to their consequent capacity to spread the traffic-induced stresses on road foundation: Flexible pavements (asphalt), rigid pavements (concrete), and semi-rigid pavements (asphalt and cement bound aggregates).

However, an additional alternative, called semi-flexible pavement, has gained much popularity over the past decades. Thanks to its characteristics, this type of pavement has been successfully applied, especially in those areas subjected to heavy and slow-moving loads [1], e.g., between 1988 and 2000, 165,000 square meters of semi-flexible pavements were constructed at Copenhagen Airport (DK).

The semi-flexible pavement’s top layer is made from Grouted Macadam, a composite pavement material made by an open-graded asphalt skeleton with 20–25% voids into which a highly flowable cementitious slurry is grouted. This hybrid material combines the flexible properties and the lack of joints of asphalt pavements with high static bearing capacity, good rut resistance, and the durability of concrete ones [2,3].

On the other hand, Grouted Macadams constitute a poorly understood branch of pavement technology and have generally been relegated to a role in certain specialty and heavy-duty pavements. This is mainly attributable to the complexity of Grouted Macadams’ construction. It is a two-stage operation, slow and expensive, and normally carried out on two consecutive days. First an open-graded asphalt layer designed to achieve a high void content is laid using a traditional asphalt paver. As soon as the asphalt has cooled, a cementitious slurry grout is spread over the surface. The filling of the voids of the asphalt is facilitated by rubber scrapers (squeegees) until the grout reaches the bottom of the layer. Moreover, the beneficial properties of this material are deeply related to the good void connectivity that allows the grout to flow through them and to an adequate workability of the grout to completely fill the voids. Unfilled voids may cause premature pavement failure.

The complexity of the laying process and the significant amount of labor required lead to long realization times and extremely high initial costs, i.e., not less than 4 times a traditional wearing course’s cost. Therefore, the use of semi-flexible pavements has been limited in its field of application and use, e.g., airport aprons, ports, and industrial storage areas, etc. [4,5].

During the last few years, an innovative material has been developed. The basic idea was to find a smart alternative to Grouted Macadam, which could be more easily employed in road pavement applications and at a lower cost.

This material, called Ready to Mix (RTM), should provide similar or even superior characteristics compared to traditional Grouted Macadam while reducing construction times and avoiding the need for dividing the laying process. 

During the development stages of the mixture, undertaken in the laboratory (the main results of the laboratory optimization phase are reported in Table 1), the potential benefits of using the RTM as a structural layer have been highlighted, and not just as an improved wearing course (such as the Grouted Macadam). Considering that the Grouted Macadam is not expected to be used as a base layer of a pavement structure, a proper comparison between Grouted Macadam and RTM cannot be undertaken, even if the Grouted Macadam represents the original inspiration of the RTM, i.e., the same type of structure coarse aggregates coated by a bituminous mortar (cold mix in the case of RTM) and bituminous film constitute the “elastic bridge” between the stones and the cement mortar.

Considering that the primary purpose of our present research activity is to evaluate the material’s properties and its resistance to dynamic load repetitions through the induced changes in field moduli and deformation trends [6].

During a first laboratory testing phase, which involved studying, characterizing, and comparing different mixtures, the optimum Ready to Mix’s composition was identified. 

This mixture is characterized by a high stiffness modulus (about 27,000 MPa, compared to the standard 12,000 MPa of Grouted Macadam) and a high resistance to dynamic load repetitions (no failure using the maximum load achievable by the asphalt standard testing machine), chemical agents, fuels, and temperatures (EN 12697-26; EN 13286-41; EN 13286-42; EN 12697-24; EN 7087). More details regarding the laboratory testing and results will be part of other papers. 

The mechanical characteristics, i.e., ITS and Resilient Modulus, of RTM are significantly higher than the Grouted Macadams’ ones (Table 1). The fatigue resistance of RTM is impressively high, while there is no information about the fatigue characteristics of Grouted Macadam, since this type of material is exclusively used as wearing course.

After this first laboratory investigation phase, wherein different mixtures have been tested and ranked, the highest performing one has been produced in a specifically designed plant, laid, and further studied by means of in-situ tests. 

The experiences reported in this paper are part of an ongoing research activity to evaluate the in-situ properties and long-term performance of the Ready to Mix mixture. The main aim of this research was a preliminary evaluation of RTM’s in-situ properties and its structural evolution. 

To evaluate the field performances of this material, two different trial sections have been designed and laid, and two different Accelerated Pavement Tests (APTs) have been carried out.

The in-situ APT tests have been undertaken using an innovative device, called Fast Falling Weight Deflectometer (FFWD). This device, which is able to apply a high number of impulsive loads in a short period of time on the pavement surface and record the deflections at different distances from the load application, is a feasible device with which to perform a quick in-field evaluation of new material.

## 2. Materials and Methods

### 2.1. Ready to Mix

Ready to Mix (RTM) is an innovative material for semi-flexible pavement wearing course. It was initially developed as a smart alternative to the traditional Grouted Macadam, which is little-used due its complexity and high cost of realization.

RTM is made by aggregates wrapped in a bituminous emulsion film and mixed with a special designed cementitious reactive filler and water, conveniently dosed to give to the mixture superior characteristics when compared to Grouted Macadam (the laboratory RTM’s composition is reported in Table 2). 

**Figure 1:**
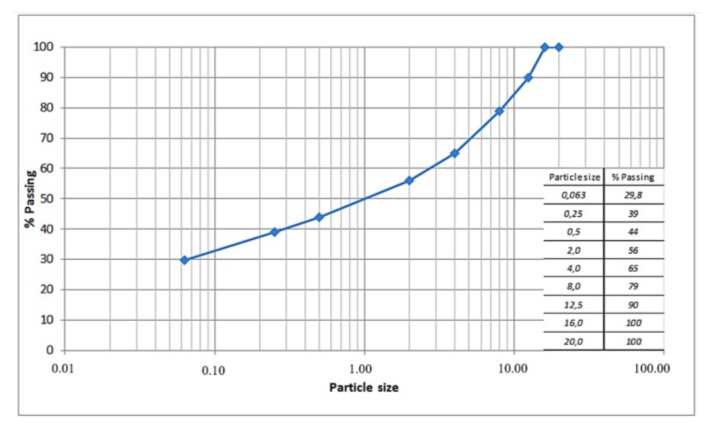
Particle size distribution curve.

The grain size distribution of the initial aggregate mixture, after the introduction of the active filler, is reported in Figure 1 and is based on the specifications provided by the Italian Road Authorities [7,8] for asphalt binder course. The same specifications were adopted also for other characteristics requested for coarse and fine aggregates. The coarse aggregates are cold mixed with a slow-setting cationic bituminous emulsion (ECL 60-type C60B4, according to EN 13808) specifically designed for this purpose. 

The cementitious grout is obtained by carefully calibrating the optimum fluid content (derived from the water added through bitumen emulsion) in combination with a specially designed reactive filler (R2M Flowflex, CVR, Gubbio, Italy), which was developed to provide the mixture with its desired characteristics: high stiffness, and thanks to the presence of the elastomeric polymers, an extremely high resistance to dynamic load repetitions (fatigue). The “R2M Flowflex” reactive filler is premixed with cement binder, polymers, and micro-silica sand. Its composition is reserved and patented. 

The percentage of reactive filler must be in the range of 30 ± 5% in weight of the total mixture, while the percentage of the bitumen emulsion is requested in the range of 4.6% ÷ 6.8%. The final percentages of the components come out from the mix design.

For both test trial sections investigated, the material has been laid via a traditional asphalt paver and dosed accurately with the correct content of water in the mixture in order to produce the right consistency. 

### 2.2. Fast Falling Weight Deflectometer-FastFWD 

Traditionally in-situ pavement response and performance can be evaluated and studied by means of Accelerated Pavement Testing (APT), which has been defined by Metcalf, as “the controlled application of a prototype wheel loading (…) to determine pavement response and performance under a controlled, accelerated accumulation of damage in a compressed time period” [9].

Accelerated Pavement Tests are able to determine pavement response and performance under a controlled, accelerated accumulation of damage, simulating field conditions. The goal of APT instrumentation is to subject an examined pavement to an extremely high number of cycles in a compressed period of time. These cycles allow for the study of the pavement’s performance evolution by monitoring the changes in deflections, moduli, and the damage growth until a failure condition is reached. These observations can be used to forecast the fatigue life of pavement structures.

In the last forty years, many full-scale APT tests have been performed to evaluate the health conditions of pavements and their performance over time in order to consequently organize and plan maintenance activities. 

Unfortunately, the accessibility to expensive full-scale APT devices is limited and facilities, budgets, staff, and time limitations can preclude the use of fixed tracks and full scale APTs. For this reason, great effort has been devoted to the development of a compact and economical structural testing device to quickly study and evaluate new material properties.

To fulfill the need of a smaller, compact, cheaper, and accessible testing device in recent years, a new generation of Falling Weight Deflectometer, called Fast Falling Weight Deflectometer (FastFWD) (Dynatest, Copenaghen, Denmark), has been developed (the main characteristics of FFWD and APT test setup are reported in Table 3).

As the FWD, it is used to measure the deflections induced by an impulsive load at different radial distances from the load application. Furthermore, the deflectometric data output can be analyzed and backcalculated through ELMOD 6 software (Dynatest, Copenaghen, Denmark). This software can be utilized to compute the effective stiffness of the pavement’s layers and to assess the remaining useful life of the infrastructure. 

The load lifting system distinguishes this device from the previously produced one (FWD). It allows one to carry out measurements at a much faster rate than the traditional FWD, i.e., up to 5 times faster per drop. The improved speed of the device and its ability to apply full scale truck wheel loads to the pavement surface suggests that the FastFWD might be a viable faster alternative to full-scale APT for pavement evaluation, to characterize pavement deformations and damage potential and to quickly evaluate new materials.

Some recent studies have shown that the FastFWD could be used as an intermediate tool between small-scale laboratory tests (bending beam, shear, and triaxial tests) and full-scale accelerated pavement tests to characterize material for road use and consequently estimate the useful life of a road pavement [10,11,12,13].

## 3. Field Tests

### 3.1. First Trial Section

#### 3.1.1. Description of the Trial Section

The primary purpose was to study the fatigue behavior of the material. To do that, the changes in field modulus trend under dynamic load repetitions have been investigated. The APT test using FastFWD was performed on April (12, 13, and 14th) of 2016. The mixture characteristics are reported in Table 4. The little differences in the mixture’s composition are imputable to the plant realization.

**Figure 2:**
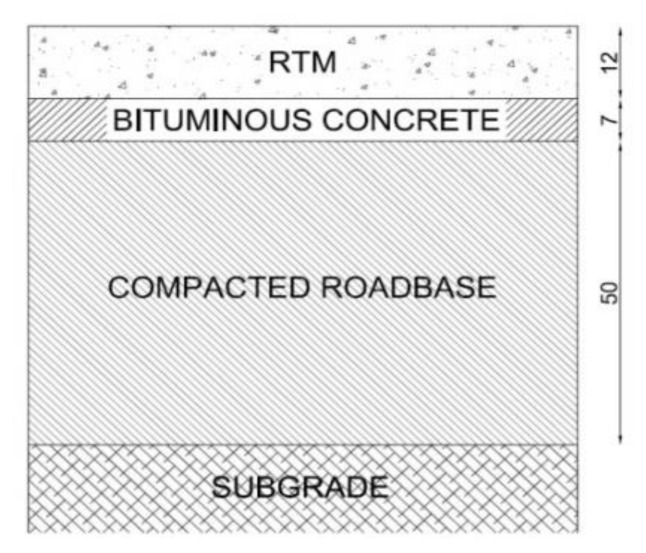
First trial section pavement’s structure.

In Figure 2, the pavement structure of the first test section is reported. It consists of four pavement layers. The top layer is a 12-cm thick layer of RTM. It has been realized on a pre-existing 7-cm thick asphalt concrete (AC) layer. The third layer is a 50-cm thick compacted roadbase laid on a cohesive subgrade.

#### 3.1.2. Test Method

In three consecutive days, a single test point has been subjected to 30,000 load cycles. The highest load level configuration of 120 kN (corresponding to a contact pressure under the loading plate of about 1600 kPa) has been applied on the pavement surface.

The FastFWD was configured with a 300-mm diameter loading plate and with nine deflector sensors, geophones, used to record vertical displacements caused by the impulsive load, positioned at 0 (under the loading plate), 200, 300, 450, 600, 900, 1200, 1500, and 1800 mm. 

The temperatures inside the RTM layer were measured hourly during the test with a probe thermometer to assess the influence of temperature variation on the test results. Air temperature and surface temperature values were also measured, stored, and compared with the RTM temperature.

Knowing the pavement structure, the recorded deflection data has been analyzed and backcalculated with ELMOD 6. The backcalculated layer modulus evolution has been plotted as the number of drops grows. 

Thanks to this backcalculation process, aimed at studying the mechanical properties of each layer of the structure, it is possible to relate and compare laboratory and field results.

Table 5 reports the FastFWD load level configuration, the contact pressure reached under the loading plate, and the number of load cycles applied on the test point for each day of the test. The first test was interrupted after 30,000 load cycles. The growth of the recorded deflections with the number of load cycles was negligible; even after applying the maximum load level configuration of the device, the strain values at the bottom of the RTM layer were small enough that it is likely that the failure condition would not be reached in a short time. 

In the first trial section, this limited number of load repetitions has no relevance for analysing long term performance of the material. Nevertheless, the recorded data are herein reported, because some fundamental properties of the material have been highlighted during this test.

#### 3.1.3. First Trial Section Results

**Figure 3:**
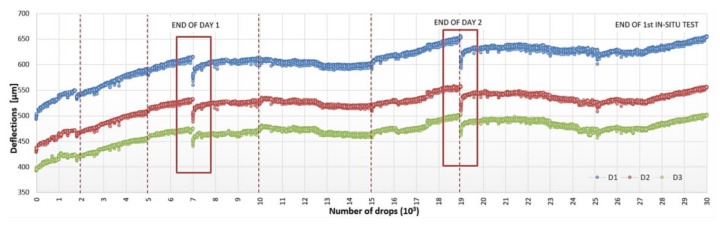
Deflections D1, D2, and D3 (recorded at 0, 200, and 300 mm from the load plate, respectively) evolution trend normalized to 1700 kPa.

**Figure 4:**
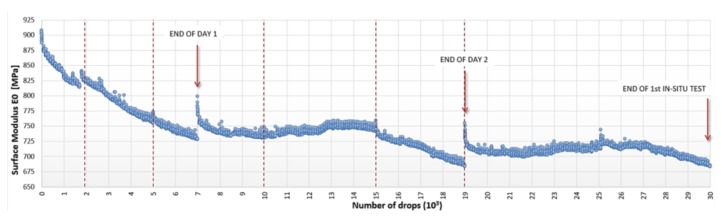
Surface moduli E_0_ evolution trend during FFWD test.

**Figure 5:**
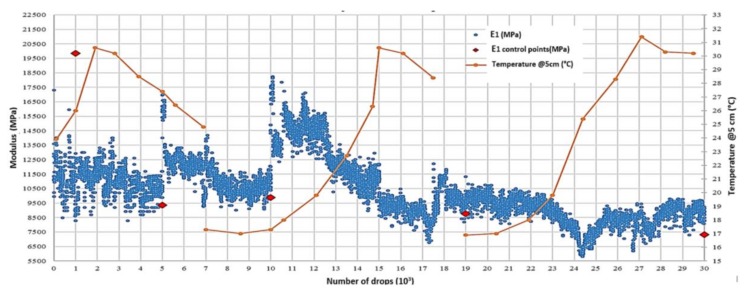
RTM modulus and temperature trends.

In Figure 3, the registered deflections trends D_1_, D_2_, and D_3_, respectively, recorded at 0, 200, and 300 mm from the loading point are reported. It is worth noting that the stiffness evolution of the surface pavement layers, the RTM layer among these, is related to the nearfield deflections [14,15] and, in particular, to their differences. This is why attention was specifically focused on the first three geophones. As the number of drops progresses, the deflection values increase. The derived pavement surface moduli E_0_ (Figure 4) decrease according to Boussinesq’s equation for a homogenous, elastic, and isotropic half-space [15]. The moduli evolution is reported in Figure 5.

To study and verify the reliability of the test procedure, the FFWD has been moved and accurately repositioned on the same test point after 2000, 5000, 10,000, 15,000, and 19,000 drops. This procedure, similarly to the Benkelman beam test method, consists of measuring the deflections produced at the center of the APT test point and moving the FFWD device in order to place each one of the geophones subsequently in the APT test point and obtaining a single deflection basin as the combination of these measurements. The comparison of this combined deflection basin with the ones measured during the APT test allows for the verification of the presence of localized cracks around the loading plate.

The discontinuities in the evolution graphs of deflections and moduli (Figure 3 and Figure 4, dashed red lines) are a consequence of checking for localized damage, stopping the APT test, moving the device to evaluate the damage, and repositioning it back on the APT test point. 

From the deflections evolution trend, it is also possible to notice that, in correspondence of the last drop in a series, the recorded deflections are bigger than the deflections caused by the first drop of the following one, e.g., in correspondence of the end of day one (N = 7000) and day two (N = 19,000). The same behavior can be observed on the derived surface moduli E_0_ evolution’s graph (Figure 4). This effect is probably due to the material’s self-healing property, which is likely attributable to the binder component of the mixture.

From the material’s moduli data output and the recorded temperatures, and referring to the Asphalt Institute’s criteria reported in Equation (1) below [16], the temperature sensibility of the RTM was investigated.

(1) $$\log_{10}E_{0}=\log_{10}E+\alpha \cdot \left(T^{2}-T_{0}^{2}\right)$$

E_0_ is the modulus, expressed in MPa, at the reference temperature T_0_. E is the backcalculated modulus, and T is the registered test temperature, both in °F. α is the corrective coefficient related to the material temperature’s sensitivity. 

From the data elaboration, the value of the RTM temperature coefficient α_RTM_ is an order of magnitude smaller than the asphalt concrete one α_AC_, as reported in the Table 6.

Moreover, it was observed that there is a negligible difference between the back calculated modulus of the RTM (Figure 5) and the RTM’s modulus normalized at the reference temperature of 20 °C (68 °F).

From these observations, it is reasonable to assume that the RTM’s stiffness is not influenced by the temperature’s effect in the range of investigated temperatures (from a minimum of 16 °C to a maximum of about 32 °C). This lack of correlation between temperature and material modulus is likely attributable to the cementitious component of the mixture.

This first in-situ experience on RTM has its limits. The presence of the asphalt concrete layer below the RTM layer leads to low strain values at the bottom of the RTM layer, and it has not been possible to quickly investigate the long-term performance of the material. A second shortcoming is imputable to the repositioning of the testing machine during the test. Hence, from the resulting data we are not able to identify how the material is able to resist repeated loading. Nevertheless, some interesting properties of the material have been highlighted, i.e., its ability to recover part of its stiffness if not stressed and its insensibility to temperature variation in the investigated temperature range. 

The second test track has been designed taking into consideration all of these key factors and shortcomings. 

### 3.2. Second Trial Section

#### 3.2.1. Description of the Trial Section

The second in-situ experience on RTM was performed on June (29, 30, and 1st of July) of 2016.

The main aim of this second field section was to reach a failure condition in a short period of time by maximizing the transverse strains at the bottom of the RTM layer. To achieve such result, unlike the first experience, a larger number of test points have been studied and the load (and the contact pressure) applied by FastFWD to the pavement surface was changed, as reported in Table 7.

#### 3.2.2. Test Method

This second in-situ test session aimed to verify the behavior of the RTM when subjected to repeated dynamic loads and working under critical stress conditions.

**Figure 6:**
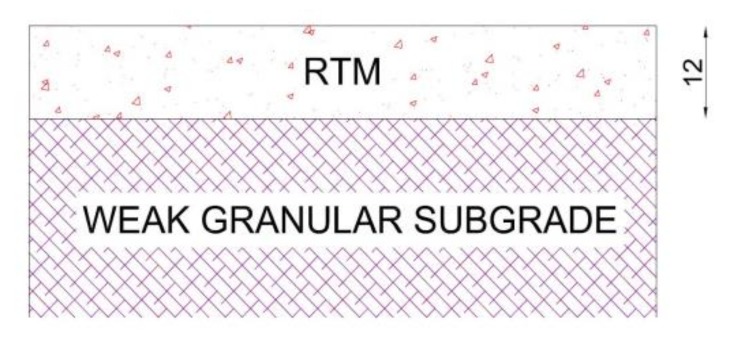
Second trial section pavement structure.

To fulfill this target, a specific field test was designed. As reported in Figure 6, the second pavement structure was made of two layers. The RTM top layer of 12 cm (around 4 inches, which is the threshold assumed to guarantee an accurate backcalculation process) [17,18] was laid on a weak pre-existing granular subgrade. This pavement structure has been designed to induce the maximum stress-strain conditions in the material and lead it to failure in a short period.

Seven different APT test points were investigated, changing the loads applied by the FastFWD to the RTM surface, reaching a total of about 60,000 drops.

Table 5 reports the load levels and the number of cycles that have been applied on each test point during this second in-situ test.

In this second case, for each investigated APT point the test has been interrupted when, in correspondence with the load plate, a deflection greater than 2000 μm (equal to the geophones’ sensibility) was reached, or when the growth of the recorded deflections when compared to the number of load cycles was negligible.

#### 3.2.3. Second Trial Section Results

**Figure 7:**
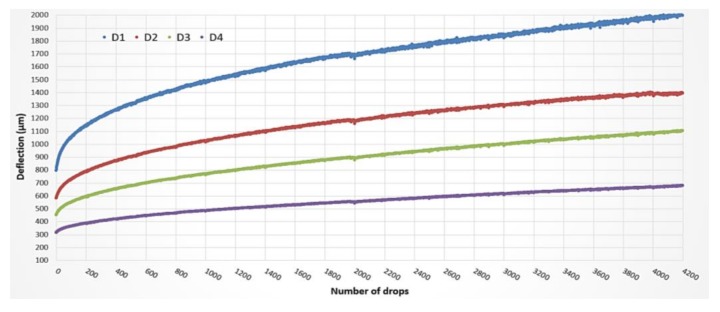
Example of recorded deflections for the test point 6.

In this second trial, the deflection graphs (Figure 7) (and surface moduli E_0_ ones) show, in each investigated point, a gradual increasing trend (decreasing for moduli ones) without irregularities. This allows confirmation that moving and repositioning the loading plate during the APT test could lead to inconsistency of results obtained from one test session to another. 

No sudden changes of slope can be observed by observing the deflection trends. 

To analyze the decay of the material’s mechanical properties, the deformation evolutions of the RTM layer have been analyzed.

Assuming as initial RTM modulus value of 32,000 MPa, defined through laboratory tests on taken cores, and as a coefficient of Poisson ν for RTM the value of 0.2, according to the cementitious characteristics of the material, the moduli decay and deformation trends at the base of the RTM layer evolution have been back calculated for each analyzed test point.

**Figure 8:**
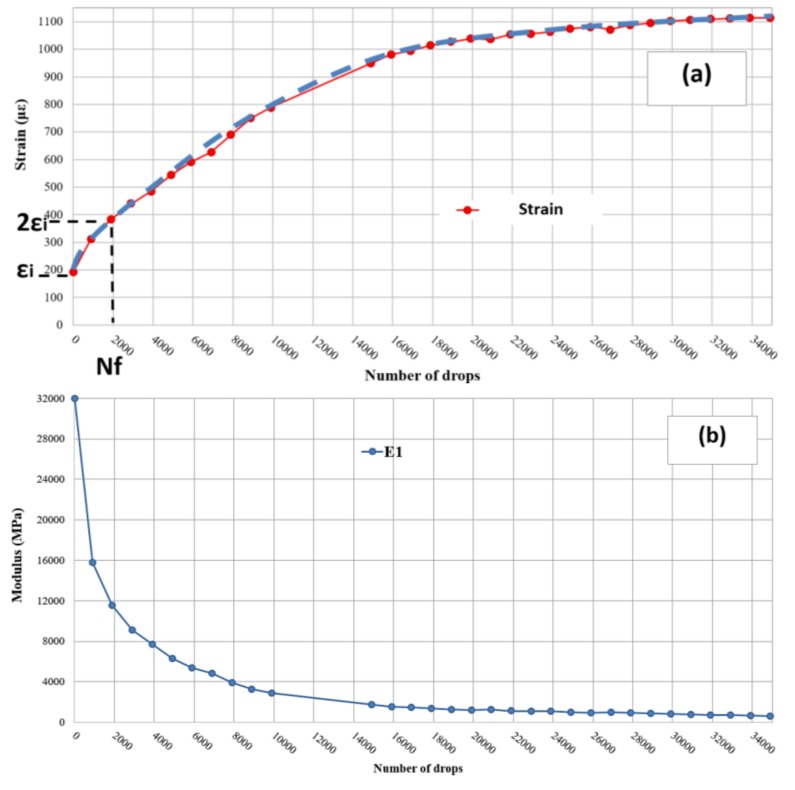
Example of backcalculated (**a**) strains and (**b**) moduli for the test point 5 (tested at 1050 kPa).

From the back calculated deformation trend of the RTM layer, a performance criterion has been identified. As pre-selected failure threshold has been assumed, the one prescribed in UNI EN 12697-24, “Bituminous Mixtures-Test Methods for Hot Mix Asphalt-Part 24: Resistance to fatigue”, is analogous to the Indirect Tensile Fatigue Test on cylindrical-shaped specimens (Annex E). The failure condition is reached when the strain at the lower interface of the RTM reaches double the initial value (Figure 8a) [19].

To compute the initial deflection, data related to the first 100 drops has been discarded, in compliance with the UNI EN standard.

Test point 3 data has been rejected because only 100 drops have been applied on the surface to reach the deflection of 2000 μm. It could be considered an anomaly.

**Figure 9:**
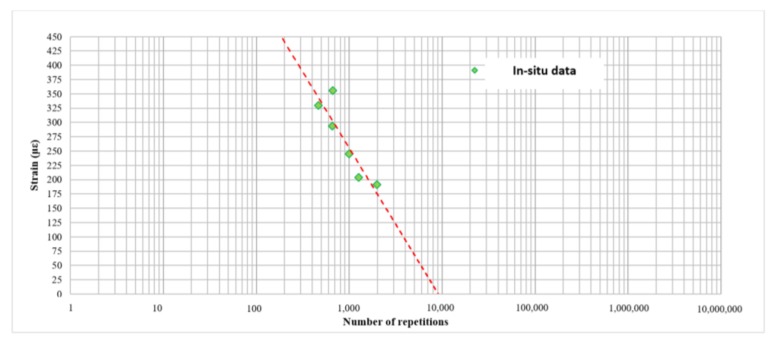
RTM performance criteria by means of in-situ data results.

In Figure 9, the RTM performance criteria is reported, based on in-situ data results. Considering this first performance criteria for low strains, the failure condition should be theoretically reached after few loading cycles. This behavior is not compatible with this type of material and with the observations that were seen during the first test session. 

To further investigate and evaluate the performance criteria of the RTM, the back calculated in field results of the seven test points have been combined with the results of the indirect tensile laboratory test IT-CY performed on cores taken from field (Table 8). 

**Figure 10:**
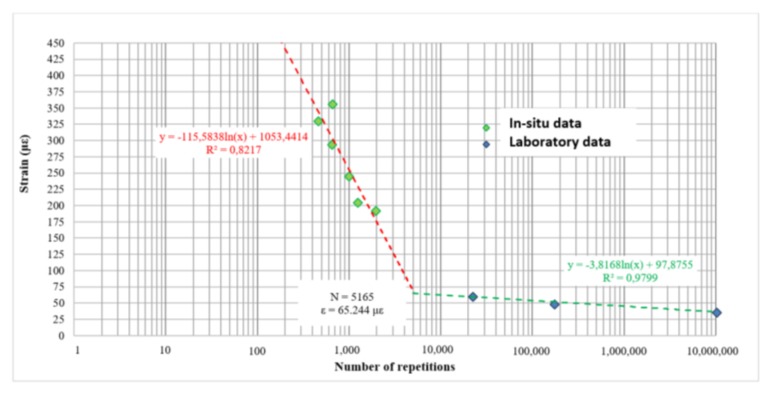
RTM performance criteria.

Thanks to the in-situ evaluation and the laboratory fatigue tests, a relation presenting the resistance to load repetition of the RTM has been defined (Figure 10).

The considered failure criterion is the same in both cases. From this point of view, the results could be considered homogeneous, even if the test conditions are significantly different, i.e. laboratory-tested samples are isolated and not confined, unlike the in-situ test points.

The obtained RTM performance criteria trend is comparable to those of cementitious materials [17], even if obtained when merging results from laboratory and site tests.

**Figure 11:**
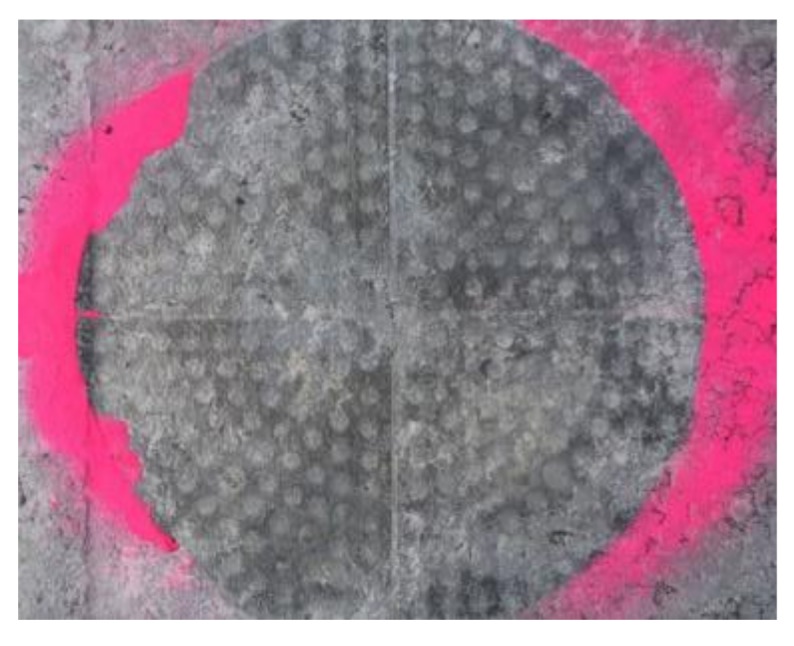
Surface of the test point 5 at the end of the test session.

Observing the surface under the APT test points at the end of each test session (Figure 11), there were no signs of cracks or permanent deformations. So, it is possible to assess that the obtained relation is quite conservative.

The self-healing properties and the material insensitivity to temperature variation have been also observed and confirmed during this second test session, e.g., self-healing can also be observed.

## 4. Discussion

The innovative material called Ready to Mix (RTM) was initially developed to be used as a semi-flexible pavement wearing course with comparable or even better characteristics and lower construction costs when compared to traditional Grouted Macadam (the Grouted Macadam costs about 9 €/sqm·cm, while RTM costs about 6.5 €/sqm·cm). During the first laboratory characterization of the material, it has shown excellent performance in terms of stiffness and dynamic load repetitions. For this reason, the material properties and performance have been investigated to evaluate the possibility of its application as a structural layer.

As an additional research activity on RTM, the resistance to repeated loads and its characteristics were investigated by means of field tests. Two different APT trial tests were carried out using the FastFWD, and the moduli evolution and surface deflections trends have been studied and evaluated. 

These tests, specifically designed and planned, have highlighted the properties and the high resistance of RTM to dynamic repeated loads, despite its high stiffness. 

During the first test session, the failure condition was never reached. Nevertheless, some observations have been made regarding the ability of the material to partly restore its stiffness after a rest period, i.e., its self-healing properties, and its temperature insensitivity. 

Thanks to the second field test, it is possible to identify a specific performance relationship that correlates the number of loads to failure with the initial deformation induced in the material by the test load. Since no evidence of cracks or permanent deformation was produced on the tested surface at the end of the surveys, the relationship can be considered conservative. 

The results of experimental field tests confirm the promising properties of the RTM when subjected to a great number of load repetitions. 

According to the preliminary technical evaluations and the cost analysis undertaken on the trial fields experience basis, this material seems to combine the best properties of the asphalt pavements (self-healing properties, durability, and the possibility to use the asphalt concrete pavers) and temperature resistance stiffness, and high resistance to point loads typical of cement concrete pavements. At the same time, it is easier and less expensive to be laid (about 25% less) by not requiring the laying process to be divided into two subsequent stages. Realization times and initial costs are consequently reduced.

Moreover, the test identified a practical performance relationship for pavement evaluation using the FFWD as an APT device. 

The investigation method presented in the paper can provide useful information regarding the long-term mechanical properties of standard or innovative materials like the one tested within the present research activity.

To reach a complete validation of the material under different loading and temperature conditions, additional research activities and a mechanistic comparison with traditional Grouted Macadam are still ongoing.

## Figures and Tables

**Table 1:** Laboratory RTM’s main results.

Compressive Strength [N/mm^2^-MPa] UNI EN 13286-41	Indirect Tensile Strenght [MPa] UNI EN 13286-42	Stiffness Modulus IT-CY UNI EN 12697-26 Annex C	Fatigue Testing IT-CY UNI EN 12697-24 Annex E
22.12	3.32	27,773	With ε = 38με NO FAILURE

**Table 2:** Laboratory RTM’s composition.

Reactive Filler [%]	Bituminous Emulsion [%]	Water [%]	Aggregates [%]
27.11	4.52	5.12	63.26

**Table 3:** Main characteristics of the FFWD and APT test setup

**Loading Plate**	**Plate Diameter** **[cm]**	**Number of Geophones**	**Loading Range [kN]**	**Load Typical Accuracy**	**Time History Window [ms]**
Segmented	30	9	7 ÷ 120	1% of reading ±1	40 ÷ 400 ms
**Geophones Meas. Range [µε]**	**Loading Time [ms]**	**Transient Load Pulse**	**Rise Time** **[ms]**	**Deflection Accuracy**	**APT Test Setup**
0 ± 2200	25 ÷ 30	Half-sine wave	10 ÷ 15	2% of reading ±2	1600 drop/h

**Table 4:** First experience RTM’s composition.

Reactive Filler [%]	Bituminous Emulsion [%]	Water [%]	Aggregates [%]
33.5	5.45	6.06	55

**Table 5:** First trial section FastFWD load levels and load cycles.

Day	Point	Load Level [kN]	Contact Pressure [kPa]	Number of Load Cycles
1	1	120	1600	7000
2				12,000
3				11,000
			Tot:	30,000

**Table 6:** Comparison between asphalt concrete (AC) and RTM temperature coefficients.

α_AC_	α_RTM_
1.474 × 10^−4^	2.645 × 10^−5^

**Table 7:** Second trial section investigated points, FastFWD load levels, and number of load cycles.

Point	Contact Pressure [kPa]	Number of Load Cycles
1	1600	1100
2	1050	10,000
3	1600	100
4	1600	400
5	1050	35,000
6	1500	4200
7	1450	8500

**Table 8:** Laboratory IT-CY test results on the cores taken from field.

Core	ε [με]	Nf	E_0_ [MPa]	E_Nf_ [MPa]
1	61	22,000	29,900	15,160
2	50	169,000	31,000	14,784
3	37	1,000,000 No failure	31,000	-
